# Challenge Dose Titration in a *Mycobacterium bovis* Infection Model in Goats

**DOI:** 10.3390/ijms25189799

**Published:** 2024-09-10

**Authors:** Elisabeth M. Liebler-Tenorio, Nadine Wedlich, Julia Figl, Heike Köhler, Reiner Ulrich, Charlotte Schröder, Melanie Rissmann, Leander Grode, Stefan H. E. Kaufmann, Christian Menge

**Affiliations:** 1 Institute of Molecular Pathogenesis, Friedrich-Loeffler-Institut, 07743 Jena, Germany; wedlichnadine@gmail.com (N.W.); julia.figl@ages.at (J.F.); heike.koehler@fli.de (H.K.); christian.menge@fli.de (C.M.); 2 Department of Experimental Animal Facilities and Biorisk Management, Friedrich-Loeffler-Institut, 17493 Greifswald, Germany; reiner.ulrich@vetmed.uni-leipzig.de (R.U.); charlotte.schroeder@fli.de (C.S.); m.rissmann@erasmusmc.nl (M.R.); 3 Serum Life Science Europe GmbH, 30659 Hannover, Germany; grode@sls-eu.com; 4 Max Planck Institute for Infection Biology, 10117 Berlin, Germany; kaufmann@mpiib-berlin.mpg.de; 5 Max Planck Institute for Multidisciplinary Sciences, 37077 Göttingen, Germany; 6 Hagler Institute for Advanced Study, Texas A&M University, College Station, TX 77843, USA

**Keywords:** tuberculosis, infection model, *Mycobacterium bovis*, goat, endobronchial inoculation, dose titration

## Abstract

Goats are natural hosts of *Mycobacterium (M.) bovis*, and affected herds can be the cause of significant economic losses. Similarites in disease course and lesions of *M. bovis* infections in goats and *M. tuberculosis* in humans make goats good models for human tuberculosis. The aim of this investigation was to characterize *M. bovis* challenge models in goats. For this, goats were endobronchially inoculated with three doses of *M. bovis* or culture medium. Clinical signs, shedding, and immune responses were monitored until 146 days post inoculation (dpi). At necropsy, lesions were examined by computed tomography, histology, and bacteriological culture. Infected goats did not develop clinical signs. *M. bovis* was cultured from feces, but never from nasal swabs. IGRAs were positive from 28 dpi onwards, antibodies at 140 dpi, and SICCT at 146 dpi. The increase in CD25^+^, IFN-γ^+^, and IFN-γ-releasing T-cell subpopulations was time-related, but not dose-dependent. All infected goats developed paucibacillary granulomas in the lungs and regional lymph nodes. *M. bovis* was regularly cultured. Dose-dependent effects included the size of pulmonary lesions, caverns, intestinal lesions, and early generalization in the high-dose group. In summary, reproducible challenge models with dose-dependent differences in lesions were established, which may serve for testing vaccines for veterinary or medical use.

## 1. Introduction

Tuberculosis (TB) remains a global health problem for humans and animals, even more than 100 years after the discovery of the causative agents, mycobacteria of the *Mycobacterium tuberculosis* complex (MTBC). In 2022, an estimated 10.6 million people fell ill with TB and 1.3 million died from it [1]. Globally, more than 50 million cattle are infected [2], but also other domestic animal species, including goats, suffer from TB. *Mycobacterium (M.) tuberculosis* is responsible for the majority of human cases [3], while tuberculosis in livestock is mainly caused by *M. bovis* and *M. caprae*, both members of the MTBC with zoonotic potential. Both species are phylogenetically closely related and differ predominantly in the presence and absence of a few regions of difference [4,5].

Among the members of MTBC, *M. bovis* has the broadest host spectrum, including livestock (cattle, goats, sheep, pigs, horses, etc.), wildlife (wild boar, deer, wild pigs, etc.), and humans [6,7,8,9]. *M. bovis* infections of animals are significant in countries in Europe [10,11,12], Asia [13,14], Africa [15], and the Americas [16,17,18]. In contrast, *M. caprae* infections seem to be restricted to continental Europe. In Spain, it is the primary cause of tuberculosis in domestic goats, posing a risk to cattle, wildlife, and humans [19,20,21]. In other European regions, such as the Alps and the Bieszczady Mountains in Poland, cross-species *M. caprae* infections are endemic, usually involving wildlife as the maintenance reservoir [22,23,24].

Despite enormous efforts, eradication campaigns against livestock TB have not been fully successful in different regions of the world. The protracted course of infection, unspecific clinical signs, and coinfections with other mycobacteria complicate the diagnosis [25]. Respiratory symptoms may occur in MTBC infections; however, many other causes are possible. Intermittent diarrhea has been reported but may also be the result of an infection with *M. avium* subsp. *paratuberculosis* (MAP) [26]. The low-grade fluctuating fever associated with TB is difficult to assess under field conditions. Eventually, infections with MTBC, MAP, and other pathogens result in emaciation and wasting [25,26].

Therefore, preventive measures, e.g., the development of effective vaccines to control infections, have become a focus of interest in recent years. Animal models are key tools in increasing our understanding of the pathogenesis and progression of disease, preclinical evaluation of new therapies, and testing of vaccine efficacy [27]. Since no model covers all aspects of infection in the target species, it is particularly advantageous if the target species can be used. Since MTBC infections cause problems in livestock, ruminant and porcine models of TB may be useful for screening prototype vaccines [28]. While cattle models have been used since the early days of research for the evaluation of vaccines against bovine tuberculosis [29], goat models have only become important in the past 15 years, predominantly as a homologous model for *M. caprae* infections.

Goats are a low-cost alternative to cattle [28] and could be a viable model not only for animal TB, but also for human TB. Progression of disease, target organs, and lesions that goats develop after infection with *M. bovis* or *M. caprae* are similar to those in humans infected with *M. tuberculosis*. The fact that goats are one of the few model species that develop cavitating pulmonary lesions that represent highly relevant lesions in human tuberculosis renders them an especially interesting model [28,30,31].

Studies in cattle have shown that dose and route of application of *M. bovis* are decisive for the manifestation of the disease. The highest efficacy of the infection route with lesions similar to field cases was achieved by intra-tracheal or aerosol administration of low doses of the pathogen (10^2^–10^3^ cfu/dose) [32]. The endobronchial route of inoculation was very effective in mimicking natural infection in experimental *M. bovis* infection models in different animal species [33,34,35] and in goat models of *M. caprae* infection. Endobronchial application of 10^3^ cfu *M. caprae* per dose resulted in an infection rate of 100% [28]. However, goats seem to be less susceptible to *M. caprae* than to *M. bovis*, because a lower total lesion score was observed after challenge of goats with 10^4^ cfu of *M. caprae* compared with 10^4^ cfu of *M. bovis* by the transthoracic route [36].

The aim of the present study was to establish and characterize infection models in goats with *M. bovis* for the testing of vaccine candidates. In an optimal model, on one hand, the infection should be highly efficient and reproducible, but on the other, it should not be overwhelming and cause excessive pulmonary lesions. This can be adjusted via inoculation parameters like strain of mycobacteria, route of application, and dose of inoculum. In the present study, infection with *M. bovis* and video-guided endobronchial spray application were used to achieve reproducibly multifocal pulmonary lesions [31]. Three different low doses of *M. bovis* were administered to discern dose-dependent reactions. A wide variety of parameters were evaluated for in-depth characterization of the models. These included systemic immune reactions in addition to clinical signs, development of lesions, and re-isolation of *M. bovis*. A particular focus was on the longitudinal characterization of IFN-γ production and secretion and upregulation of activation marker CD25 by T-cell subpopulations in response to MTBC antigens, because novel vaccine candidates were designed to optimize the response of certain T-cell subpopulations [37].

## 2. Results

### 2.1. Clinical Investigation of M. bovis-Inoculated Goats

Regular examinations did not reveal any differences between *M. bovis*-inoculated and mock-inoculated goats in terms of general condition, appetite, impulsive coughing, or gain of body weight. Concerning body temperature, an increased individual fluctuation range was observed in all infected goats (Figure S1), although the mean rectal temperatures of all groups were within the physiological range reported for goats (38.5–39.5 °C) [38]. This fluctuation was particularly marked between 14 dpi and 50 dpi. In the further course of the study, the group that had received the high dose of inoculum (HD-group) was most severely affected (Figure S1). After intradermal application of PPDs for the SICCT at 143 dpi, the rectal temperature rose significantly in some of the infected goats.

Some clinical signs were seen in all groups, including the mock-inoculated controls, and were not related to the inoculation with *M. bovis.* These included provoked coughing for three weeks after the intrabronchial inoculation, occasional spontaneous coughing, and sporadic changes of fecal consistency throughout the study. One goat that later received the high dose of inoculum developed intermittent diarrhea of unknown cause 14 days prior to inoculation; this goat lost appetite and decreased in general condition till the end of the study.

### 2.2. Results of Intra Vitam Testing for Indications of Mycobacterial Infection

**Figure 1:**
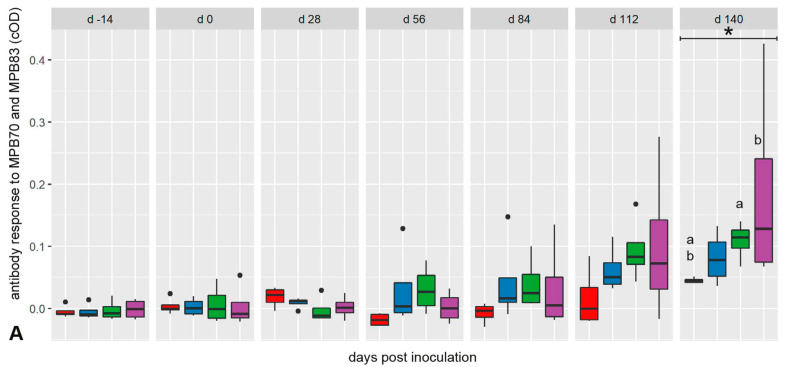

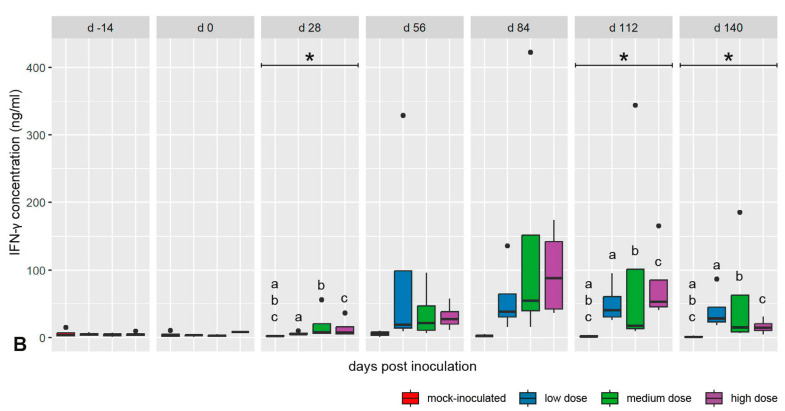
(**A**) Time course of the antibody response against mycobacterial antigens (MPB70 and MPB83) in *M. bovis*-inoculated and mock-inoculated goats. Serum samples were tested with a modified IDEXX *M. bovis* antibody ELISA. Significant differences between infected and mock-inoculated goats were detected only at 140 dpi. (**B**) Time course of the antigen-specific IFN-γ release response of whole blood samples after re-stimulation with bPPD. Significant differences between infected and mock-inoculated goats were detected at 28 dpi, 112 dpi, and 140 dpi. Box–whisker plots represent median, 25%, and 75% quartiles, lowest and highest values and outliers as dots for n = 4 animals per group. Dose groups are color-coded. Significant group differences detected by the Kruskal–Wallis test (*p* ≤ 0.05) are labeled with an asterisk, significant differences detected by pairwise comparison with the Mann–Whitney U test (*p* ≤ 0.05) with identical lower-case letters.

The antibody response against antigens MPB70 and MPB83, specific for mycobacteria of the MTBC, developed slowly after infection (Figure 1A). Differences between mock-inoculated animals, the group that had received the medium dose of inoculum (MD group), and the HD group, respectively, became significant at 140 dpi. The inoculation with *M. bovis* also elicited an antigen-specific adaptive T-cell response, which preceded the humoral response. Independent of the inoculation dose, an increased bPPD-induced IFN-γ release response was detectable at 28 dpi in all inoculated groups (Figure 1B). Values peaked at 84 dpi and remained different from the mock-inoculated goats until 140 dpi.

**Figure 2:**
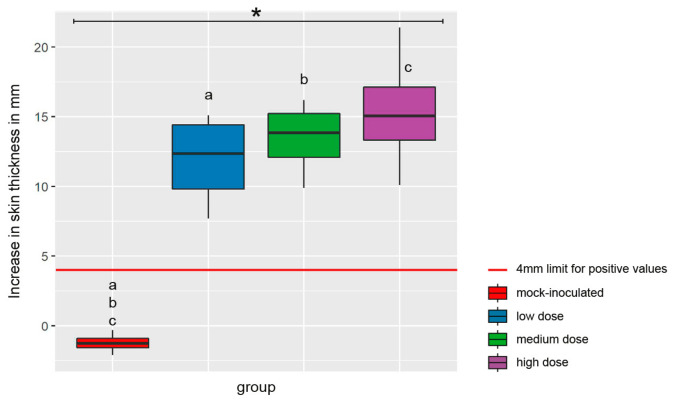
Results of the intradermal skin test (SICCT) 146 days after intrabronchial inoculation of goats with *M. bovis*. The test is considered positive if the increase in skin fold thickness after application of bPPD is ≥4 mm more than after application of aPPD (red line). Box–whisker plots represent median, 25%, and 75% quartiles and the lowest and highest values for n = 4 animals per group. Dose groups are color coded. Significant group differences detected by the Kruskal–Wallis test (*p* ≤ 0.05) are labeled with an asterisk, significant differences detected by pairwise comparison with the Mann–Whitney U test (*p* ≤ 0.05) with identical lower-case letters.

A significant SICCT response was recorded at 146 dpi for all *M. bovis*-inoculated goats (Figure 2). The increase in skin fold thickness after application of bPPD did not depend on the inoculation doses.

### 2.3. Quantitation of M. bovis Antigen-Specific T Cells After In Vitro Re-Stimulation

**Figure 3:**
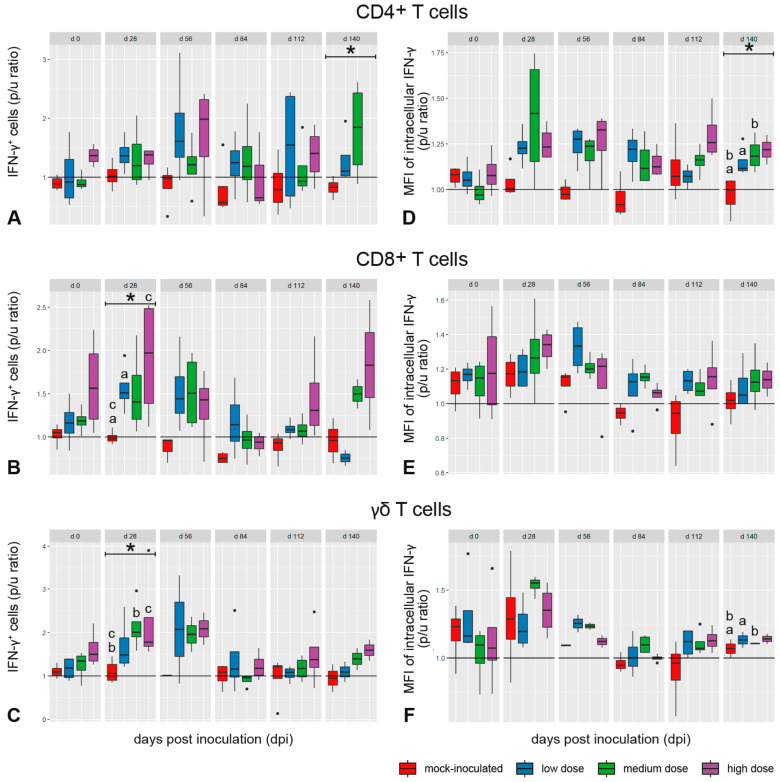
Percentage of IFN-γ^+^ T cells (**A**–**C**) and median fluorescence intensity (MFI) of intracellular IFN-γ (**D**–**F**) in CD4^+^ (**A**,**D**), CD8^+^ (**B**,**E**), and γδ (**C**,**F**) T-cells from PBMC after re-stimulation with *M. bovis* antigen in vitro, normalized as p/u ratio (bPPD stimulated cells/unstimulated cells). Significant increases in IFN-γ expression were detected in CD8^+^ and γδ T cells 28 dpi, and intracellular IFN-γ was increased in CD4^+^ T cells at 140 dpi. Box–whisker plots represent median, 25%, and 75% quartiles, lowest and highest values and outliers as dots for n = 4 animals per group. Dose groups are color coded. Significant group differences detected by the Kruskal-Wallis test (*p* ≤ 0.05) are labeled with an asterisk, significant differences detected by pairwise comparison with the Mann-Whitney-U test (*p* ≤ 0.05) by identical lower-case letters.

Proportions of CD4^+^, CD8^+^, and γδ T cells in peripheral blood mononuclear cell (PBMC) cultures did not differ between animal groups throughout the experiment. In vitro stimulation of PBMCs with bPPD increased the proportion of IFN-γ^+^ cells in CD4^+^, CD8^+^, and γδ T cell subsets of all *M. bovis*-inoculated groups (Figure 3A–C). Despite considerable individual variation, a marked proportional increase compared with the mock-inoculated group was observed in all studied lymphocyte subsets at 28 dpi and 56 dpi, and primarily in CD4^+^ T cells again at 140 dpi. This was independent of the inoculation dose.

A marked increase in the abundance of intracellular IFN-γ after bPPD stimulation in vitro, expressed as median fluorescence intensity (MFI)*,* was observed in CD4^+^ T cells between 28 dpi and 84 dpi, and to a lesser extent in CD8^+^ T cells between 56 dpi and 112 dpi in the inoculated goats (Figure 3D,E). A specific response compared with the mock-inoculated group was not observed for the γδ T cells. Differences between the inoculated groups were not noted (Figure 3F).

**Figure 4:**
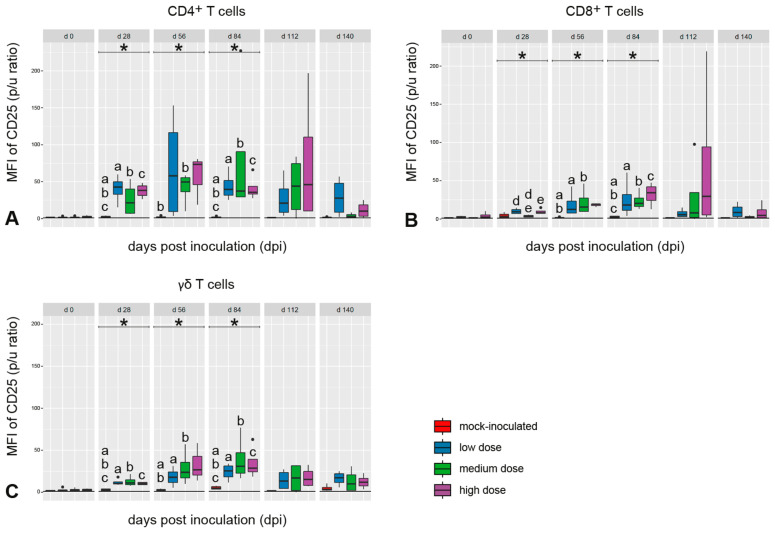
Median fluorescence intensity (MFI) of CD25 on the surface of CD45RO^+^ T cells, analyzed for CD4^+^ (**A**), CD8^+^ (**B**) and γδ (**C**) T cells from PBMC after re-stimulation with *M. bovis* antigen in vitro. There was a transient increase in all T-cell subsets from 28 dpi to 84 dpi. Box–whisker-plots represent median, 25%, and 75% quartiles, lowest and highest values and outliers as dots for n = 4 animals per group. Dose groups are color coded. Significant group differences detected by the Kruskal–Wallis test (*p* ≤ 0.05) are labeled with an asterisk, significant differences detected by pairwise comparison with the Mann–Whitney U test (*p* ≤ 0.05) with identical lower-case letters.

The expression of CD25 in CD45RO^+^ cells belonging to the CD4^+^, CD8^+^, or γδ T-cell subsets was significantly upregulated in the *M. bovis*-inoculated goats after in vitro stimulation of PBMC with bPPD between 28 dpi and 84 dpi (Figure 4). The expression levels peaked between 28 dpi and 84 dpi in these goats without clear group differences and decreased later on with large individual variation. No distinct CD25 expression was measured in these cell populations in the mock-inoculated animals (Figure 4).

### 2.4. Shedding of the Inoculum Strain via Nose and Feces

*M. bovis* was not shed to the environment by respiratory secretions of the goats. Occasional shedding was detected in the feces of animals of the MD and HD groups from 56 dpi onwards.

### 2.5. Computed Tomography Imaging (CT) of Lungs from M. bovis-Inoculated Goats

**Figure 5:**
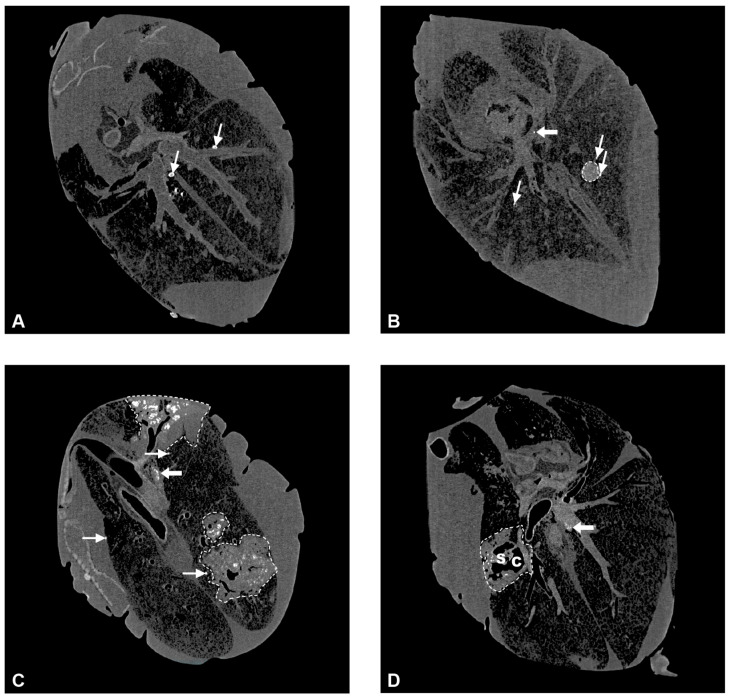
Computed tomography scans of lungs from the *M. bovis*-inoculated goats. (**A**) Examples of multiple micronodules (MNs, thin arrows) in the right and left basal lobe of a goat from the LD group (goat 6). Most MNs are located adjacent to airways. (**B**) Example of a unicentric consolidation >5 mm (delineated by a hatched line) in the right basal lobe of a goat from the MD group (goat 9). MNs are present adjacent to the UC and in the left basal lobe (thin arrows). A lesion in a tracheobronchial LN is marked with a thick arrow. (**C**) Example of three multicentric consolidations (delineated by hatched lines) in the right cranial and basal lobes of a goat from the HD group (goat 15). Several MNs are present in addition (thin arrows). Lesions in the cranial tracheobronchial LN are marked with a thick arrow. (**D**) Example of a cavern (C) partitioned by septae (S) in the center of a multicentric consolidation (delineated by a hatched line) in the left basal lobe of a goat from the HD group (goat 14).

CT allowed determination of total lung volume, total number of lesions, total lesion volume, and thus, the percentage of lung volume affected by lesions (Table S1). Hyperdense lesions presented as round micronodules (MNs) less than 5 mm in diameter with smooth borders and a density of >200 Hounsfield units (HUs), indicating mineralization, and larger, round to pleomorphic consolidations with diameters of more than 5 mm and variable patterns of mineralization (Figure 5A–C). These patterns were used for further subdivision of lesions as (1) unicentric, mostly round consolidations (UCs, Figure 5B), which were partly or diffusely mineralized and sometimes dust-like, or (2) multicentric consolidations (MCs, Figure 5C) with multiple foci of mineralization and a predominantly irregular shape. In the center of some MCs, hypodense areas matching the air density were present. If their diameters were larger than the airways in this region and they had irregular outlines, they were diagnosed as caverns (Figure 5D).

**Figure 6:**
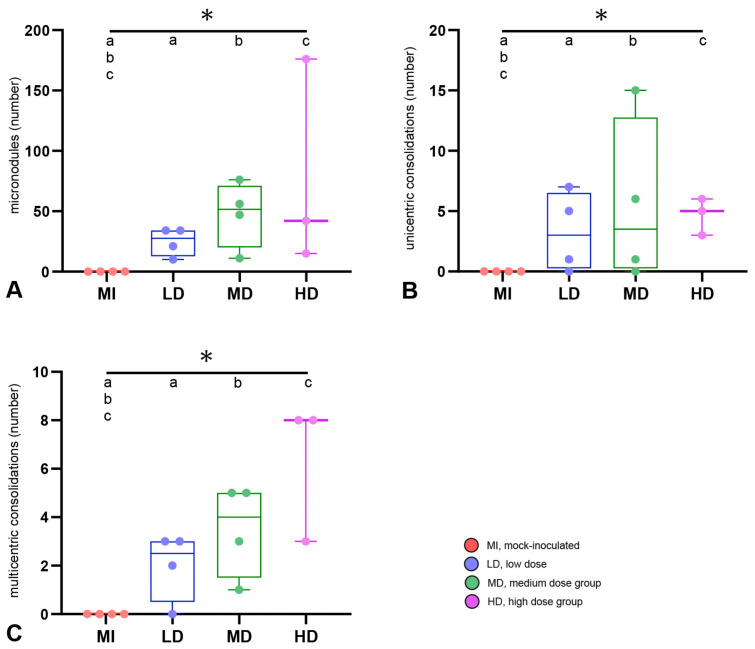
Number of lesions detected by computed tomography imaging. (**A**) Micronodules (small < 5 mm). (**B**) Unicentric consolidations (>5 mm, focal mineralization). (**C**) Multicentric consolidation (>5 mm, multiple mineralizations). n = 4 animals per group, except for HD group (n = 3); box–whisker plots represent medians, 25%, and 75% quartiles, the lowest and highest values, and counts of individual goats. Significant group differences detected by the Kruskal–Wallis test (*p* ≤ 0.05) are labeled with an asterisk, significant differences detected by pairwise comparison via the Mann–Whitney U test (*p* ≤ 0.05) with identical lower-case letters.

**Figure 7:**
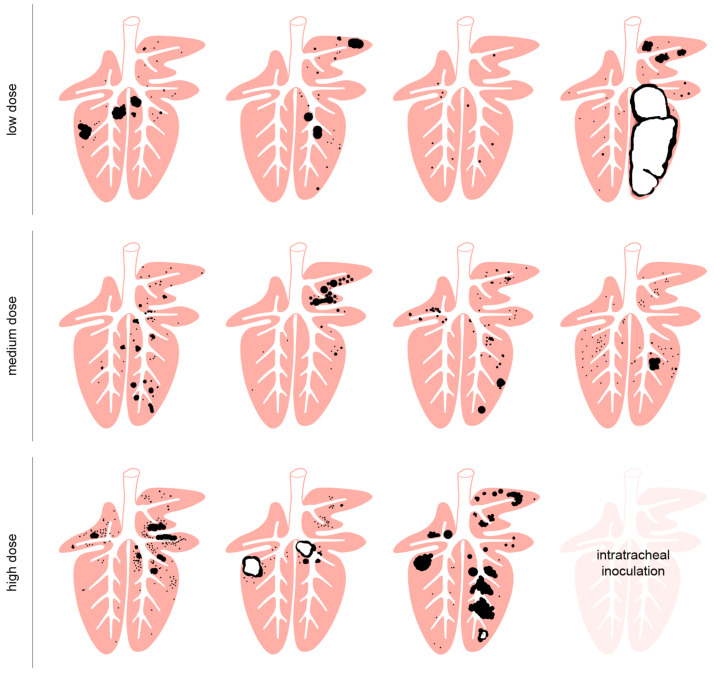
Graphical visualization of size and location of pulmonary TB lesions detected by computed tomography imaging. Micronodules and consolidations (black) and caverns (black with white centers) are represented as spheres of the measured volume and located at the site where they were identified in the individual goats of the LD, MD, and HD groups. No pulmonary lesions were detected by computed tomography imaging in the intratracheally inoculated goat.

**Figure 8:**
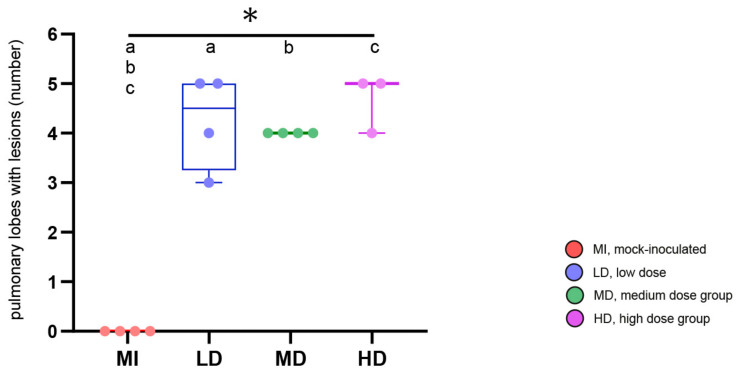
Number of pulmonary lobes with lesions. Groups are color-coded with 4 animals per group, except for HD group (n = 3); box–whisker plots represent medians, 25%, and 75% quartiles, the lowest and highest values, and counts in individual goats. Significant group differences detected by the Kruskal–Wallis test (*p* ≤ 0.05) are labeled with an asterisk, significant differences detected by pairwise comparison via the Mann–Whitney U test (*p* ≤ 0.05) with identical lower-case letters.

All goats that had received *M. bovis* by endobronchial inoculation developed hyperdense lesions in the lung (Figure 6), whereas none of these lesions were detected in the controls or in the goat with intratracheal inoculation, which was therefore excluded from further analysis of CT data. The frequency of all types of lesions was significantly different between the *M. bovis*-inoculated groups and the mock-inoculated controls, but not between the different dose groups when groups were compared pairwise via the Mann–Whitney U test (Figure 6). The type and number of hyperdense lesions varied between and within inoculation groups (Figure 7). Lesions were widely distributed throughout the lung, affecting the majority of lobes independent of the inoculation dose (Figure 8), and were generally associated with airways.

**Figure 9:**
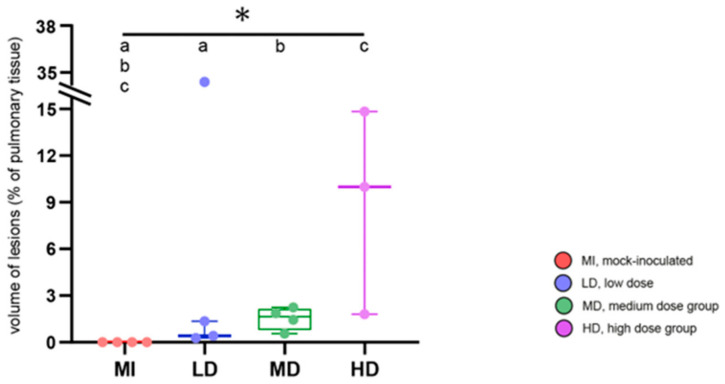
Volume of pulmonary lesions as percentage of lung volume. Groups are color-coded with 4 animals per group, except for HD group (n = 3); box–whisker plots represent medians, 25%, and 75% quartiles, the lowest and highest values, and data for individual goats. Significant group differences detected by the Kruskal–Wallis test (*p* ≤ 0.05) are labeled with an asterisk, significant differences detected by pairwise comparison via the Mann–Whitney U test (*p* ≤ 0.05) with identical lower-case letters.

In the group that received the low dose of inoculum (LD-group), one goat had only MNs, three had MNs, UCs, and MCs, and one had caverns (Figure 6 and Figure 7). MNs were present most frequently (mean 25, range 10–34), followed by UCs (mean 3, range 0–7) and MCs (mean 2, range 0–3, Table S1). The volume of lesions was comparable in three goats and much larger in one goat, resulting in percentages of altered pulmonary tissue ranging from 0.28% to 34.31% (Figure 9, Table S1). The latter was the highest percentage of altered pulmonary tissue encountered in the *M. bovis*-inoculated goats, caused by extensive MCs and caverns throughout the right basal lobe. In the MD group, all goats had MNs, UCs, and MCs, but no caverns were detected (Figure 6 and Figure 7). As in the LD group, MNs were most frequent (mean 48, range 11–76), followed by UCs (mean 6, range 0–15) and MCs (mean 4, range 1–5, Table S1). Although the numbers of lesions were not significantly different between individuals from the LD and MD groups, average numbers were higher in the MD group. The volume of lesions was on average 42.3 cm^3^ (range 12.8–66.1 cm^3^), resulting in an average percentage of altered pulmonary tissue of 1.53% (range 0.56–2.24%, Figure 9, Table S1). In the HD group, the three endobronchially inoculated goats had MNs, UCs, and MCs (Figure 6 and Figure 7). Caverns were detected in two goats in the basal lobes only. As in the LD and MD groups, MNs were most frequent (mean 78, range 151–176), followed by MCs (mean 6, range 3–8) and UCs (mean 5, range 3–6) which was on average an increase compared with the MD dose group (Table S1). The volume of lesions was on average 200.3 cm^3^ (range 39.7–463.1 cm^3^), resulting in an average percentage of altered pulmonary tissue of 6.66% (range 1.81–14.83%, Figure 9, Table S1). The percentage of lung volume affected by lesions was significantly different between the *M. bovis*-inoculated groups and the mock-inoculated controls, but not between the different dose groups (Figure 9).

Multiple foci of mineralization were detected in tracheobronchial and pulmonary LNs of all *M. bovis*-inoculated but none of the mock-inoculated goats (Figure 5, Table 1). The cranial tracheobronchial LNs were most frequently and most severely affected, whereas lesions were present in the pulmonary LNs of one or two goats per group only.

### 2.6. Macroscopic and Histologic Lesions, and Detection of Acid-Fast Bacilli in Tissues from M. bovis-Inoculated Goats

**Figure 10:**
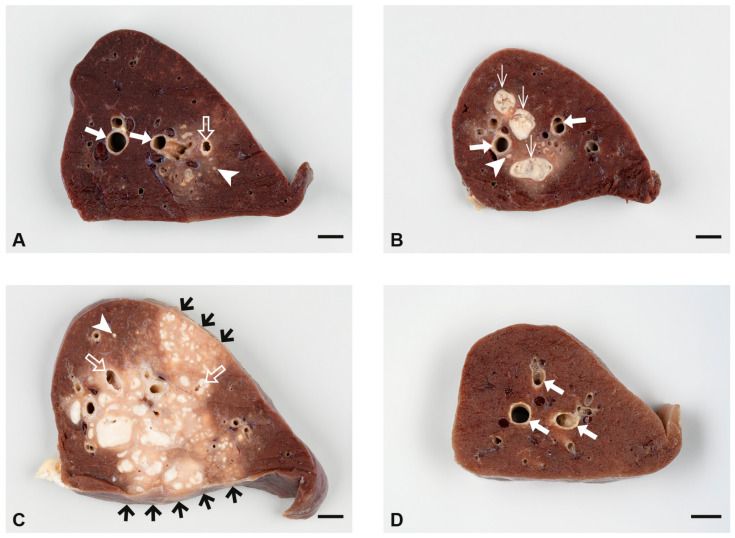
Sections of formalin-fixed caudal lung lobes of one representative goat per group inoculated with LD (**A**), MD (**B**), and HD (**C**) of *M. bovis* and of a mock-inoculated goat (**D**). (**A**) Small granulomas (arrowhead) and a cavern (open arrow); larger bronchi are indicated by filled arrows, goat 7. (**B**) Small granulomas (arrowheads, examples) and granulomas larger than 5 mm (thin arrows) with central caseous necrosis (white) next to large bronchi (arrows), goat 9. (**C**) A large area of confluent granulomas of variable size (light colored) with multiple foci of caseous necrosis (white) has replaced most of the pulmonary tissue (dark brown) and extends multifocally to the serosal surfaces (short black arrows). Small granulomas are present in the adjacent pulmonary tissue (arrowheads, examples). Two caverns are indicated by open arrows, goat 15. (**D**) Unaltered pulmonary tissue with large bronchi indicated by filled arrows, goat 4. Size bars = 1 cm.

Lungs and LNs draining the lung were predominantly affected by lesions in all goats of the LD, MD, and HD groups, but in none of the mock-inoculated goats (Table 2). Granulomas characteristic of TB with central necrosis and mineralization were additionally present in the lungs and LNs of infected goats and caverns in the lungs. Granulomas were classified as type 1 (groups of epithelioid cells and multinucleated giant cells), type 2 (type 1 granuloma with minimal necrosis), type 3 (monocentric granuloma with central necrosis and mineralization), or type 4 (multicentric granuloma with central necrosis and mineralization) according to Wangoo et al. (2005) [39]. Dose-dependent differences occurred in respect of severity and extent of pulmonary lesions (Figure 10). Overall, type 1 and 2 granulomas with minimal necrosis became apparent by histology only, while type 3 and 4 granulomas were already grossly recognized. The central necrosis of type 3 and 4 granulomas was surrounded by an inflammatory cell infiltrate including epithelioid cells and multinucleated giant cells, and a fibrotic capsule of variable thickness. Intact and degraded neutrophils were frequent in the transition zone of necrosis and inflammatory cell infiltrate. Pulmonary caverns were cavities surrounded by necrosis and an inflammatory cell infiltrate as seen in type 3 and 4 granulomas. Acid-fast bacilli (AFB) were rarely detected.

In the LD group, pulmonary lesions were noted in the lungs of all goats (Table 2). In three goats, low numbers of small granulomas (1 mm to 5 mm) and small caverns were located deep in the pulmonary tissue close to the main bronchi (Figure 10A). Histology showed type 1 to 4 granulomas and caverns frequently associated with bronchi, bronchioli, and bronchus-associated lymphoid tissue (BALT). AFB were found only in one goat at the edge of a cavern. The fourth goat of the LD group had a severely enlarged right caudal lobe attached to the thoracic pleura by chronic fibrous pleuritis and numerous granulomas throughout the lung. Dissection of the lung confirmed the complete replacement of the right caudal lobe by a large cavern surrounded by confluent granulomas. Despite the extensive inflammatory infiltrates and necrosis, no AFB were detected.

All goats of the LD group developed granulomas (predominantly types 3 and 4) in the mediastinal and tracheobronchial LNs. Single AFB were detected only in granulomas of the goat with the severe pulmonary lesions. This animal was the only one of the LD group with moderate thymic atrophy and dissemination of mycobacteria to gut-associated lymphoid tissue (GALT), causing type 1 granulomas, as well as to mesenteric and ileocolic LNs, causing type 1, 2, and 3 granulomas.

Pulmonary lesions were present in all goats of the MD group (Table 2). In one goat, granulomas and caverns were located deep in the pulmonary tissue close to the main bronchi only (Figure 10B), while in three goats, lesions were already visible at the surface of the lung, variably affecting different lung lobes and often associated with circumscribed chronic fibrous pleuritis. In two goats, confluent granulomas occurred in addition to individual small lesions. Compared with goats of the LD group, lesions larger than 1 cm were more frequent. Histological examination revealed type 1 to 4 granulomas, while AFB were not detected.

Granulomas comparable to those of the LD group occurred in the tracheobronchial LNs of all four goats and in the mediastinal LNs of two goats (Table 2). Few AFB were found in the tracheobronchial and mediastinal LNs of one goat only. Dissemination was observed in two goats: one had a type 3 granuloma with numerous satellite granulomas in the liver; another one had multiple type 1 granulomas in a Peyer`s patch in the jejunum and a Peyer`s patch in the ileum (Table 2).

In the HD group, extensive pulmonary lesions were seen in the three goats that were inoculated by the endobronchial route (Table 2, Figure 10C). Two goats had chronic adhesive pleuritis associated with pulmonary lesions and one of them several small granulomas in the pleura costalis at the adhesion sites. Histological examination revealed type 3 to 4 granulomas and caverns in the confluent lesions and type 1 and 2 granulomas in the surrounding pulmonary tissue. AFB were not detected. In the goat inoculated by intratracheal injection, extensive type 1 to 3 granulomas were present at the injection site and the tracheal mucosa was ulcerated. There were, however, only a few, small pulmonary lesions. AFB were not detected.

All goats of the HD group developed granulomas in tracheobronchial and mediastinal LNs comparable to those in the LD and MD groups. A few AFB were detected in granulomas in the tracheobronchial and mediastinal LNs of two goats.

Dissemination was most extensive in the three goats of the HD group with endobronchial inoculation (Table 2). Granulomas were seen in the palatine tonsil, retropharyngeal LNs, liver, spleen, and various sites of GALT, as well as mesenteric and ileocolonic LNs. AFB were found in granulomas in the GALT or mesenteric LNs of two goats. No dissemination was observed in the goat with intratracheal inoculation.

All *M. bovis*-inoculated goats developed severe local tissue reactions and activation of the draining of superficial cervical LNs at the site where bPPD was injected for SICCT. The local reaction to aPPD was mild in the LD and MD groups and moderate in the HD group. No tissue reactions were detected in the mock-inoculated goats.

### 2.7. Dissemination of the Inoculum Strain in the Body

*M. bovis* was culturally recovered from tissues collected at necropsy of 11 of the 12 inoculated goats, but not from 1 goat of the MD group (Table 2). *M. bovis* was isolated from the mediastinal LNs of 10 goats and from the left tracheobronchial LN of 8 goats. Dissemination of *M. bovis* to the retropharyngeal LNs, tonsils, or mandibular LN was sporadically seen in individual goats of all inoculation groups. Likewise, *M. bovis* was isolated from the Peyer’s patches and draining LNs of the jejunum or ileum of individual goats of all three groups. Dissemination to parenchymal organs (liver, spleen, or hepatic lymph node) was observed only in two animals of the HD group. For technical reasons (requirement of fixation for CT imaging), pulmonary tissues were not subject to cultural isolation.

## 3. Discussion

Subclinical TB was reproducibly induced in goats that received low doses of *M. bovis* by video-guided endobronchial spray application. *M. bovis* was chosen as inoculum since it is overall more widely distributed in ruminants compared to *M. caprae*, but both mycobacteria induce comparable lesions [28,30,40,41,42]. A German field isolate was selected, because circulating field strains of a pathogen are required in challenge models for vaccine testing and licensing. The course of the disease and the presence of lesions predominantly in the respiratory tract observed in our experimental infection corresponded with field cases of TB in goats caused by *M. bovis* [9,43,44] and experimental infections [45,46]. *M. tuberculosis*, which is the most important cause of TB in humans, was not used as inoculum, because it causes only mild lesions in goats [36].

A problem with the goat model of tuberculosis is the induction of severe locally extensive pulmonary lesions, which complicate the evaluation of vaccine effects [28]. Intranasal application has been attempted in order to overcome this problem but resulted in severe lesions in the upper respiratory tract [47]. Low doses of *M. bovis* were applied in the present study. Re-titration of aliquots of the inocula revealed that the intended doses of 5 × 10^1^ cfu, 5 × 10^2^ cfu, and 5 × 10^3^ cfu had to be corrected and 4.71 × 10^2^ cfu, 8.85 × 10^2^ cfu, and 8.28 × 10^3^ cfu had actually been given. Thus, the effects of two rather close doses (LD and MD) and a higher dose (HD) were compared. This confirms the difficulties in the titration of mycobacteria due to their tendency to aggregate and clump [48]. The doses used for inoculation were comparable with 10^2^ cfu of *M. bovis* for intratracheal inoculation [45] and 10^3^ cfu and 10^4^ cfu of *M. bovis* given by aerosol [46].

The course of infection was clinically inapparent in all dose groups. Manifestations like loss of condition, drop in milk yield, and coughing—as seen in some field cases of caprine TB and experimental infections with *M. caprae*—did not occur [28,43,44,45,49]. This may have been due either to the young age of the goats in our study, the short observation period of about 5 months, or optimal husbandry conditions without requirement of performance which enabled the animals to compensate for the pulmonary lesions and other foci of inflammation without clinical signs. Daily assessment of body temperature revealed an increased day-to-day fluctuation in infected goats, indicating host–pathogen interactions, but no significant overall increase as described after experimental infection with *M. caprae* was seen [28]. The fluctuation was more pronounced in the HD group, reflecting more severe pulmonary lesions and more frequent systemic spread in this dose group. The temperature curve was similar to the low-grade intermittent fever reported in human cases of TB [50,51]. The group differences in body temperature observed did not, however, allow unequivocal identification of infected goats. Thus, the clinical parameters recorded in this study were not suitable to monitor the progression of infection.

A range of specific immune reactions were evaluated for their potential to monitor the infection intra vitam. As has been shown previously, T-cell responses preceded humoral responses by several weeks [28]. IGRA results allowed confirmation of infection as early as 28 dpi. This was in line with observations in other experimental settings using higher infectious doses, where IFN-γ responses were detected between 15 dpi and 30 dpi [47]. Antibody responses were already measurable in individual goats from 56 dpi onwards. Due to the low numbers of animals per group, a significant increase was detected only at the end of the observation period. Other authors have also reported antibody responses at about 100 dpi [28] or a variable onset of antibody responses depending on the antigens used for detection [47]. The SICCT performed at the end of the trial was positive in all *M. bovis*-infected goats, confirming its good correlation with the presence of TB lesions. In field outbreaks, its usefulness for detecting infected herds and controlling TB infection has been emphasized [9,43,52]. Neither IGRA, nor AB titers, nor SICCT were influenced by the inoculation doses applied.

Further dissection of specific T-cell responses revealed no expansion of distinct T-cell subsets during infection, confirming findings after experimental infections of goats using *M. caprae* [53] but contrasting with findings in *M. bovis*-infected cattle, where the CD4–CD8 ratio varied over time [54]. The proportions of IFN-γ^+^ CD8^+^ and γδ T cells were increased in infected animals at the time when the IGRA became positive. Although the proportion of IFN-γ^+^ CD4^+^ T cells was not significantly elevated at that time, intracellular IFN-γ was most strongly upregulated in CD4^+^ T cells. The proportion of IFN-γ^+^ T cells and the strength of its intracellular expression in the T-cell subsets followed an intermittent course until the end of the study. IFN-γ expression in CD4^+^ T cells has been reported as reliable marker of infection [53] and it has been suggested that the number of antigen-specific IFN-γ-secreting effector CD4^+^ T-cells is correlated with the severity of disease [55].

IFN-γ reactivity increased synchronously with an increase in CD25^+^ expression in all T-cell subsets upon re-stimulation with mycobacterial antigen, which was interpreted as in vitro reactivation of non-naïve T cells, since CD25, which is the α chain of the high affinity receptor for IL-2, is upregulated in activated T cells [56]. T cells with a memory phenotype (CD45R0^+^) expressing CD25 and CD26 were identified as the predominant cell type responding to mycobacterial antigens in calves infected with *M. avium* spp. *paratuberculosis* [57]. Quantitation of the responses by flow cytometry was considered a consistently reliable way to monitor the evolution and changes in the immune response occurring during mycobacterial disease progression. In our study, data obtained by this method revealed differences between *M. bovis*-infected versus mock-inoculated goats more consistently between 28 dpi and 84 dpi than quantitation of IFN-γ-producing T-cell subsets, implying that the former approach is also better suited for monitoring the development of a specific cellular immune response in *M. bovis*-infected goats. Although regulatory T cells (T regs) were not addressed in this study, it cannot be ruled out that a fraction of the CD25^+^/CD4^+^ T cells were T regs, which counterbalance pro-inflammatory responses and limit tissue damage [58]. Higher numbers of T regs in the blood were correlated with less severe pulmonary lesions in non-human primate models of TB [59]. The lower numbers of CD25^+^ T cells at the end of the experiment, paralleling a decline in IFN-γ release in PBMC cultures from *M. bovis*-inoculated goats, may indicate that these reactions were transient, possibly because proliferation of *M. bovis* was limited by local immune responses, granuloma formation, and the low numbers of AFB within lesions.

Nasal shedding was not detected in any of the *M. bovis*-infected goats, although the inoculum had been placed endobronchially and even endotracheally in one goat. Even in goats that had pulmonary caverns or tracheal ulceration, which are generally considered as indications for “open” TB, no excretion of mycobacteria via nasal secretion was detected. This confirms field studies where positive nasal swabs were found in less than 10% of *M. bovis*-exposed goats [60]. A possible explanation might be that coughing was rarely observed in our goats and thus, infectious material from pulmonary and tracheal lesions may have been swallowed and passed at the laryngopharyngeal junction to the digestive tract. The delayed onset of fecal shedding detected sporadically between 56 dpi and 140 dpi argues against swallowing of mycobacteria during the inoculation procedure, but rather from pulmonary lesions. The higher frequency of fecal shedding in goats from the HD-group correlates with the more extensive pulmonary lesions and more frequent caverns in this dose group. On the other hand, mycobacteria in the feces might have originated from intestinal lesions which were also more frequent in the HD-group.

Different infection routes, e.g., transthoracic [36,61], endobronchial [28,31,47], intranasal [47], by aerosol [46], and by contact with infected goats [62,63], have been assessed with the endobronchial route emerging as the best compromise between applicability, reproducibility and type, severity and organ distribution of TB lesions. This was confirmed in the present trial: the endobronchial inoculation induced pulmonary lesions characteristic for TB in all goats irrespective of the dose applied [30,44,52,64]. As has been shown, the combination of CT, macroscopic, histological and microbiological analyses allowed a comprehensive evaluation of the pulmonary lesions [28,47,49]. This revealed both dose-independent and dose-dependent findings. Numbers of micronodules, distribution of lesions in the majority of pulmonary lobes and presence of granulomas of all stages were not dose-dependent. This was most likely an advantage of the mode of application by spraying the inoculum at multiple sites of the lung and avoiding deposition of boli of inoculum [31]. In contrast, multicentric consolidations were more frequent in the HD-group compared to the LD- and MD-groups and thus, there were also higher numbers of micronodules in close association with these consolidations. Micronodules in close association with consolidations which are also referred to as “daughter” micronodules are considered to be important indicators for the progression of TB in non-human primates [58,65]. The dose dependence of caverns remained unresolved, because no dose-dependent differences occurred if small caverns in the size of bronchioles and bronchi were included, whereas large caverns were more frequent in the HD-group. Dose-dependent differences were also observed in the volume of lung affected by lesions, with larger volumes in the HD-group. The volumes in the LD- and MD-group were lower than the means of 3.8% and 5.3% reported after endobronchial inoculation with 1.5 × 10^3^ cfu of *M. caprae* [28,49]. These dose-dependent differences did not reach the level of significance, because of the small number of animals and the marked individual variation in the HD-group.

Documentation of pulmonary lesions for each individual goat showed the extend of this variation between individuals even within dose-groups. It also revealed the completely different development of infection in two of the goats. One goat in the LD-group developed very severe and extensive pulmonary lesions. Since this goat had the lowest social position in its group and was harassed by the other goats, the permanent stress might have influenced its immune responses, although no clear indications of immunosuppression were detected. One the other hand, the goat in the HD-group that received the inoculum intratracheally developed marked local lesions and only minor pulmonary lesions. Comparable local lesions have been described after intratracheal inoculation, but in addition to pulmonary lesions [45]. These findings underline the importance of sufficiently large animal groups for treatment or vaccination efficacy testing.

All infected goats developed a complete primary complex, i.e., TB lesions in the lung as primary organ and in the regional LNN, as has been reported in field infections [43,44,66]. In contrast to pulmonary lesions, those in the regional LNN were not dose-dependent.

There were two further dose-dependent effects. Lesions in gut-associated lymphoid tissues and/or the draining mesenteric LNN were observed in single goats of the LD- and MD-group, but in all goats of the HD-group after endobronchial inoculation. This was frequently associated with TB granulomas in tonsils and LNN of the upper digestive tract and isolation of *M. bovis* from these tissues which suggests that infectious material derived from the lungs was swallowed and passed along the digestive tract. The frequent location of lesions in gut-associated lymphoid tissue as result of the preferential uptake of mycobacteria by M cells in follicle-associated epithelium has been shown in ruminants [67,68]. Dissemination to spleen and liver by the lympho-hematogenous route as early generalization was also more frequent in the HD-group. Both forms of dissemination were also frequently reported in field infections [9,43,66,69].

Cultural isolation of *M. bovis* was possible from most of the lesions, although AFB were rarely detected in tissue sections independent of the inoculation dose. Variable and often low numbers of AFB have been reported in TB of goats [9,43,66]. In one goat of the LD-group, AFB were detected in the inner lining of a cavern; this has been explained as increased mycobacterial growth caused by the elevated oxygen tension at this site [66,70,71]. Cultural isolation of *M. bovis* was also possible from sites without lesions. This higher sensitivity of cultural isolation in comparison with histology may be due to the fact that larger amounts of tissues are used for cultivation.

Overall, a reproducible model of subclinical primarily respiratory TB with granulomas and caverns was established in goats. The individual variations observed stress the necessity of sufficiently high numbers of individuals per group for comparison of treatment effects which can be easier achieved with goats than cattle. The occurrence of dose-dependent and dose-independent effects allows to choose an appropriate model for treatment or vaccine testing by adjusting the dose of the inoculum.

## 4. Materials and Methods

### 4.1. Animals, Housing Conditions and Nutrition Regime

Sixteen two-week-old male goats of the breed “German Improved White” were purchased from a conventionally kept herd of dairy goats with no history of bovine TB. They were raised in the experimental animal rooms of the Friedrich Loeffler Institut, Jena, Germany (FLI Jena) as described previously [31]. At the age of six months, goats were transferred to the biosafety level 3 animal facility at the FLI site Greifswald—Insel Riems, Germany (FLI Riems). Upon arrival, animals were divided randomly into four groups of four goats. They were accommodated for two weeks before first sampling and four weeks before endobronchial inoculation with *M. bovis*. Continuing with the same diet (hay cobs, water ad libitum, occasionally vegetables), husbandry was realized in air-conditioned loose boxes with negative air-pressure and artificial daylight, on rubber mats, with enrichment consisting of wooden branches for nibbling and inverted mortar buckets for climbing [31].

### 4.2. Legislation and Ethical Approval

This study was carried out in strict accordance with European and National Law for the Care and Use of Animals. The protocol was reviewed by the Committee on the Ethics of Animal Experiments of the State of Mecklenburg–Western Pomerania, Germany and approved by the competent authority (permit number: 7221.3-1-084/16, date of permission: 8 February 2017). The infection experiment was conducted in a contained area at biosafety level 3 under supervision of the authorized institutional Agent for Animal Protection. Bronchoscopy was performed strictly under general anesthesia as described below. During the entire study, every effort was made to minimize the animals’ suffering.

### 4.3. Inoculum and Inoculation

*M. bovis* strain SB0989 (collection of the German National Reference Laboratory for Bovine Tuberculosis, FLI Jena) was cultured in Middlebrook 7H9 (MB) bouillon for four months, characterized by real-time PCR targeting IS*1081*, and the colony count determined [72,73]. The bacterial suspension was cryo-conserved in MB bouillon supplemented with glycerin and an antibiotic mixture containing polymyxin B, amphotericin B, nalidixic acid, trimethoprim, and azlocillin and stored at −80 °C. For inoculation, the strain was defrosted and transferred into phosphate buffered saline (PBS). The suspension was centrifuged at 4000× *g* for ten minutes at 20 °C; the pellet was resuspended in 1 mL sterile PBS and further diluted with PBS to achieve the intended bacterial counts per dose.

Quantitative re-assessment of remaining inoculum yielded 4.71 × 10^2^ cfu per animal for the LD group, 8.85 × 10^2^ cfu for the MD group, and 8.28 × 10^3^ cfu for the HD group. In a preceding experiment [31], the colony counts of the bacterial suspension before and after passage through a spray catheter had been determined to ascertain that there was no marked loss of bacteria. Quality control of the inoculum after use, according to endpoint and real-time PCR using the targets IS*1081* [74] and RD4 [75], as well as by spoligotyping [73] and MIRU-VNTR [76,77], confirmed that the inoculum contained *M. bovis* lineage BOV_1 SB0989, MIRU-24 profile 222324253322645431223433.

**Figure 11:**
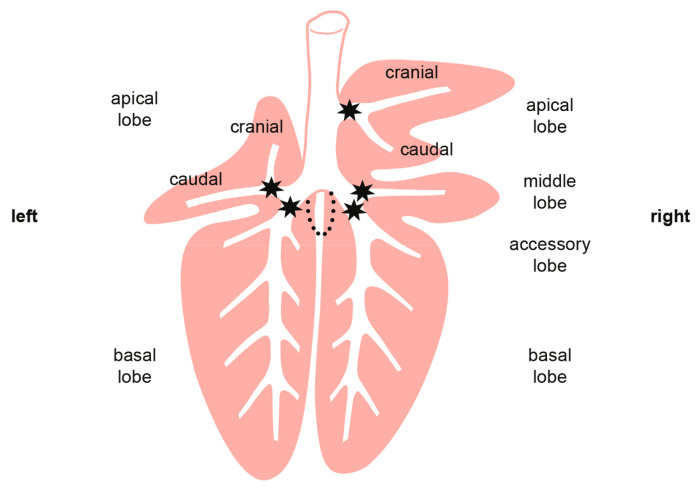
Pulmonary sites (stars) where inoculum was deposited by spray catheter.

Goats were endobronchially inoculated under deep sedation as described previously [31]. Each animal received 2.5 mL of a defined dose of *M. bovis* suspension (n = 4 goats in LD and MD groups, n = 3 goats in HD group), or 2.5 mL PBS for the mock-inoculated group (n = 4). The suspension was administered at five defined locations (500 µL each) in the lung, deploying a spray catheter (KARL STORZ GmbH & Co. KG, Tuttlingen, Germany) that was inserted through the working channel of the endoscope. Positions for application were the bronchus trachealis, the bronchi principales dexter et sinister, the bronchus lobaris cranialis, and the bronchus lobaris medialis (Figure 11) [31]. Due to technical problems, one goat of the HD group (n = 1) was inoculated by injecting 2.5 mL of the *M. bovis* suspension directly into the trachea.

### 4.4. Clinical Examination and Intra Vitam Sampling

A comprehensive clinical examination of the animals was carried out daily. Rectal temperature was measured. The breath rate was counted. General condition, skin and hair, eye and nasal discharge, conjunctiva and oral mucosa, breathing patterns, spontaneous and inducible coughing, appetite, rumination, legs, and peripheral lymph nodes were scored (Table S2) and the weekly averages calculated and used for statistical evaluation. Body weight was measured in weekly intervals.

Blood was obtained as ethylenediaminetetraacetic acid (EDTA) blood, heparinized blood, and serum from the external jugular vein 14 days prior to inoculation, on the day of inoculation, and every four weeks after inoculation. Nasal swabs and fecal samples were collected for mycobacterial culture at the same time points and additionally daily during the first 14 dpi.

### 4.5. Intra Vitam Testing for Indications of Mycobacterial Infection

Serum antibodies against *M. bovis* were detected with a modified IDEXX *M. bovis* antibody ELISA (IDEXX, Westbrook, ME, USA), in a test designed to detect antibodies against MPB70 and MPB83. The IgG HRP conjugate of the kit was replaced with the anti-ruminant IgG HRP conjugate from an IDEXX Paratuberculosis Screening ELISA (IDEXX, Montpellier, France). Except for the mentioned modification, the test was performed following the manufacturer’s instructions.

For the interferon gamma release assay (IGRA), heparinized blood was stimulated overnight with 300 IU bPPD/mL (Prionics Lelystad B.V., Lelystad, The Netherlands) or with 250 IU/mL aPPD (Prionics Lelystad B.V., Lelystad, The Netherlands). Pokeweed mitogen-stimulated blood (5 µg/mL) served as positive control, blood complemented with cell culture medium only (RPMI 1640 W/GLUTAMAX I, Sigma Aldrich Chemie GmbH, Taufkirchen, Germany) as negative control. IFN-γ in supernatants was quantified by in-house capture ELISA using monoclonal antibodies against bovine IFN-γ as previously described [78].

A SICCT was performed three days prior to necropsy, at 143 dpi. Into the right side of the neck, 0.1 mL (5000 IU) of bPPD (WDT, Garbsen, Germany) was injected intracutaneously. Into the left side of the neck, 0.1 mL (2500 IU) of aPPD (WDT, Garbsen, Germany) was applied. Skin fold thickness was recorded prior to PPD injection and about 72 h after injection at 146 dpi. Application and analysis were performed according to European Commission’s tuberculosis regulations (annex B to council directive 64/432/EEC).

### 4.6. Quantitation of Intracellular IFN-γ in CD4^+^, CD8^+^ and γδ T Cells after In Vitro-Stimulation with Specific Antigen

Antigen-specific T-cell responses were monitored by means of IFN-γ production of T-cell subsets after in vitro exposure to specific antigens following a described protocol [78]. In brief, PBMCs were isolated by density gradient centrifugation, adjusted to a final cell count of 2 × 10^6^ per ml and incubated for 21 h at 37 ± 2 °C. Cells were either stimulated with 300 IU/mL bPPD (Prionics Lelystad B.V., Lelystad, The Netherlands) or left unstimulated. Concanavalin A (1 µg/mL, Merck, Darmstadt, Germany)-stimulated cultures served as positive controls. For the last 3 h of incubation, phorbol 12-myristate 13-acetate (PMA, 50 ng/mL, Merck), ionomycin (1 µg/mL, Merck), and brefeldin A (1 µL/mL, Golgi Plug^TM^, BD Biosciences, San Jose, USA) were added. For fluorescence staining, anti-CD4 (clone GC1A, Kingfisher Biotech, Saint Paul, MN, USA), anti-CD8α (MCA2216GA, Biorad, Hercules, CA, USA), and anti-γδ TCR1-N24 δ chain (clone GB21A, Kingfisher Biotech) were used as the primary antibodies. After incubation with primary antibodies, PBMCs were fixed and permeabilized with BD Cytofix/Cytoperm (BD Biosciences). This was followed by staining with secondary antibodies (anti-mouse IgG_2a_ Alexa fluor 633 [Southern Biotech, Birmingham, AL, USA], anti-mouse IgG_2b_-FITC [Southern Biotech], and directly PE-labelled anti-IFN-γ [clone CC302, Kingfisher Biotech]). Samples from 0 and 28 dpi were analyzed with FACS CANTO II (BD Biosciences), whereas subsequent samples (56 dpi until 140 dpi) were analyzed with MACS Quant Analyzer 10 (Milteny Biotec, Bergisch Gladbach, Germany). Data analysis was performed with Flow Jo (BD Biosciences). The applied gating strategy has been described before [75]. Gates and quadrants to distinguish cells with specific signals from auto-fluorescent cells were set according to the negative control incubated with secondary antibodies, defining only less than 2% of the cells as positive. Median fluorescence intensities for the detection of IFN-γ were recorded as the signal for the detection of PE for all cells gated for CD4, CD8α, and γδ TCR1-N24 positivity, respectively. As a measure of the antigen-specific effect for all analytes, the p/u ratio was calculated as the quotient of the results (percent positive cells or median fluorescence intensity for all cells of the subset) for bPPD-stimulated cells and the results of unstimulated cells.

$$\left(\frac{\%\;or\;MFI\;\;bPPD\;stimulated\;cells}{\%\;or\;MFI\;unstimulated\;cells}\right)$$

### 4.7. Quantitation of Antigen-Specific Peripheral T Cells by Activation Marker Expression Analysis after In Vitro Stimulation with Specific Antigens

**Figure 12:**
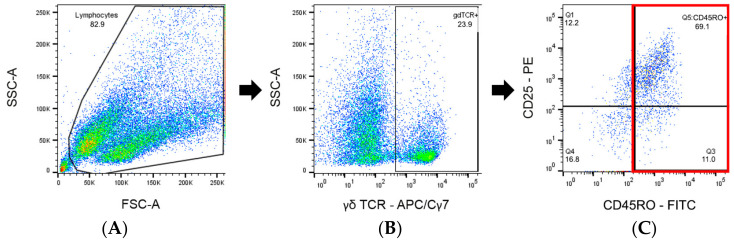
Gating strategy for quantitation of antigen-specific peripheral T cells by activation marker expression analysis (example). (**A**) Gating for live and morphologically intact lymphocytes. (**B**) Gating for the γδ TCR^+^ population. (**C**) Gating for CD45RO^+^ T-cells from the γδ TCR^+^ population. The median fluorescence intensity (MFI) for CD25 (MFI CD25 (γδ TCR^+^/CD45RO^+^).

PBMCs were isolated and stimulated as described above. All cultures (bPPD-stimulated, concanavalin A-stimulated, and unstimulated) were incubated for 5 days at 37 ± 2 °C. Then, cells were harvested and stained with the following primary antibodies: anti-CD45RO (clone IL-A116, Biorad), anti-CD25 (clone CACT116A, Kingfisher Biotech), anti-CD4 (clone GC50A1, Kingfisher Biotech), anti-CD8α (clone CC63, Biorad), anti-γδ TCR1-N24 (clone GB21A, Kingfisher Biotech). Anti-mouse IgG_3_ human adsorbed FITC (Southern Biotech), F(ab’)_2_ anti-mouse IgG_1_ human adsorbed PE (Southern Biotech), anti-mouse IgG_2a_ Alexa fluor 633 (Southern Biotech), anti-mouse IgG_M_ human adsorbed PE/Cy7 (Southern Biotech), anti-mouse IgG_2b_ human adsorbed APC/Cy7 (Southern Biotech) were used as secondary antibodies. The gating strategy and analysis of samples and data were analogous to the previous paragraph. Cells were gated for membership of one of the major subsets (CD4^+^, CD8^+^, or γδ TCR^+^ cells) and for the expression of detectable amounts of CD45RO on their surface (Figure 12). For all gated cells, the fluorescence signal for the dye used to detect CD25 was documented as median fluorescence intensity (MFI) without distinguishing between CD25^+^ and CD25^−^ cells. Obtained values are referred to as MFI CD25 (CD4^+^/CD45RO^+^), MFI CD25 (CD8^+^/CD45RO^+^) and MFI CD25 (γδ TCR^+^/CD45RO^+^), respectively, throughout the manuscript. The p/u ratio was calculated as described above.

### 4.8. Euthanasia, Necropsy, Gross Pathology, Tissue Sampling

Goats were euthanized by intravenous injection of 100 mg/kg pentobarbital sodium (Release 500 mg/mL^®^, WDT, Garbsen, Germany) after prior sedation by intramuscular injection of 0.2 mg/kg Xylazin (Rompun^®^ 2%, Provet AG, Lyssach, Switzerland) at 146 dpi. Necropsies were performed with macroscopic assessment, preparation of lungs for CT, and sampling of tissues for histological and bacteriological examination.

Tissues sampled included the following: injection site of PPDa, injection site of PPDb, left and right superficial cervical LNs, left and right mandibular LNs, left and right parotid LNs, tonsils, left and right medial retropharyngeal LNs, left tracheobronchial LN, mediastinal LNs, thymus, heart, liver, spleen, kidney, renal LNs, bone marrow, Peyer’s patches in jejunum and ileum, mesenteric LNs, and ileocolic LNs. An aliquot of each tissue was fixed for histological examination in 4% NBF; another aliquot was collected for cultural isolation of mycobacteria under sterile conditions.

### 4.9. Radiological Examination by Computed Tomography (CT)

Lungs were fixed intrathoracically in situ by instillation of 1–2 L of 4% NBF [31]. After removal from the thoracic cavity, superficial lesions were assessed, and formalin-instilled lungs were kept intact for further CT examination. Lungs were stored for two months in 3–4 L of 4% neutral buffered formalin (NBF) at 4° C. NBF in the storage containers was renewed every month. In preparation for scanning by CT, lungs were removed from the containers and the NBF was drained. Lungs enclosed in plastic bags inside a plastic container were scanned by CT as described [31]. Data were analyzed using Synedra personal view© version 23.0.0.1 (x64 CommunityEdition), synedra AIM version 23 “Selene” and 3D Slicer© version 4.11.20210226. Lesions were categorized into three classes (micronodules < 5 mm [MN], unicentric consolidations > 5 mm with focal mineralization [UC], and multicentric consolidations > 5 mm with multiple foci of mineralization [MC]) as detailed in the results section. The volume of the altered lung tissue was determined, both automatically and with the freehand tool of the 3D Slicer© version 4.11.20210226. For the automatic measurement, the threshold function was used with the range set from 250 to maximum Hounsfield units (HUs). Freehand measurement was used for small or less mineralized lesions, which were not completely recognized by the threshold function. These lesions were marked with the freehand tool in every slice. The software enabled calculation of the lesion volume according to the automatic and freehand measurements. When CT examination was completed, lungs were dissected and sampled for histologic examination.

### 4.10. Histologic Examination

Tissues for histologic evaluation were embedded in paraffin. Sections were stained with hemalaun and eosin (HE) for overall morphologic assessment. Granulomas were classified as type 1 (groups of epithelioid cells and multinucleated giant cells), type 2 (type 1 granuloma with minimal necrosis), type 3 (monocentric granuloma with central necrosis and mineralization), or type 4 (multicentric granuloma with central necrosis and mineralization) [39]. Ziehl–Neelsen (ZN) staining was performed for acid-fast bacteria (AFB). The number of AFB within caseous necrosis and inflammatory infiltrate of granulomas was scored as few (+, <10% of cells with few AFB and/or few/focal AFB in the necrosis), many (++, 10–50% of cells with few AFB or 10–30% of cells with many AFB and/or multifocal small groups of AFB in the necrosis), or numerous (+++, >50% of cells with few AFB or >30% of cells with many AFB and/or numerous, multifocal to diffuse AFB in the necrosis).

### 4.11. Bacterial Examination of Feces, Nasal Swabs and Tissue Samples

Feces, nasal swabs, and tissues collected at necropsy (Table 2) were examined by mycobacterial culture as described [78]. Cultures were checked every two weeks for colony growth. Once visible colonies appeared, presence of *M. bovis* was confirmed by real-time PCR targeting IS*1081* [79] and by endpoint PCR targeting RD4 [72]. When *M. bovis* could not be confirmed in the colony material, mycobacterial species identification was carried out via conventional PCRs targeting IS *1245* and *901* for members of the *M. avium* complex [80,81] and IS *900* for *M. avium* subsp. *paratuberculosis* [82]. Mycobacterial species not identifiable by these PCRs were identified by nucleotide sequence analysis (GATC, Konstanz, Germany) of a PCR-generated DNA fragment of the 16S rRNA gene [83].

### 4.12. Statistical Analysis

The Kruskal–Wallis test by rank was used first to detect significant differences between dose groups. As a nonparametric test, it did not require a normal distribution of measurements. The Kruskal–Wallis test is very powerful for detecting overall differences between treatment groups, but does not identify the exact groups that differ. When the Kruskal–Wallis test indicated significant differences between the dose groups, the nonparametric Mann–Whitney U test to compare only two treatment groups was applied as a subsequent test for all group combinations. A significant difference in medians between the two groups examined can be interpreted as a significant Mann–Whitney U test result. A significance level of 5% (*p* ≤ 0.05) was chosen for both tests. Statistics were calculated using R versions R 4.1.1 (R Foundation, Vienna, Austria) and SPSS version 19.002 (IBM Deutschland GmbH, Ehningen, Germany) and MedCalc version 19.4 (MedCalc Software, Ostende, Belgium). Data were plotted as box–whisker plots to show the median (horizontal line), the interquartile range (box), and minimum and maximum (whiskers). Values beyond 1.5 times the interquartile range were depicted as outliers (dots), but were included in the statistical analyses.

## 5. Patents

S.H.E.K. and L.G. are co-holders of a patent on the tuberculosis vaccine, VPM1002, licensed to Vakzine Projekt Management GmbH, Hannover, Germany and Serum Institute India Pvt. Ltd., Pune, India.

## Figures and Tables

**Table 1:** Lesions in pulmonary lymph nodes detected by CT scans as hyperdense lesions or by macroscopic inspection as mineralized granulomas.

Group	Goat	Cranial Tracheo-Bronchial LNs	Left Tracheo-Bronchial LNs *	Right Tracheo-Bronchial LNs	Mid Tracheo-Bronchial LNs	Pulmonary LNs
low dose	5	++	+++	+++	-	-
	6	+++	++	-	++	-
	7	+++	-	-	+++	-
	8	+++	+++	+++	++	+
medium dose	9	++	+	+	+	-
	10	+++	++	-	-	+
	11	+++	++	++	-	++
	12	+++	+	-	-	-
high dose	13	+++	++	++	++	-
	14	++	++	+++	-	+
	15	+++	+++	+++	++	-

- no lesions; + mild/multiple small 1–2 mm lesions; ++ moderate/multiple up to 5 mm lesions; +++ extensive/lesions throughout entire LN; * lesions were detected by macroscopic inspection.

**Table 2:** Distribution of lesions characteristic of tuberculosis and cultural isolation of *M. bovis* in lymphatic tissues and parenchymal organs of the *M. bovis*- and mock-inoculated goats.

Group	Goat	Head LNs and Tonsils							Respir. Tract			Large Parenchyma							Gastrointestinal Tract					Other Sites
		1	2	3	4	5	6	7	8	9	10 ^A^	11	12	13	14	15	16	17	18	19	20	21	22	23
control	1 to 4																							
low dose	5								+	⊕	+													
	6								⊕	⊕	+												O	
	7								⊕	⊕	+													
	8					O		O	⊕	⊕	+									O	⊕	⊕	⊕	
medium dose	9					O	O		⊕	O	+								⊕	⊕		O	O	
	10								⊕		+	⊕												
	11								⊕	⊕	+													
	12								+	+	+													
high dose	13								⊕	⊕	+			⊕								+		
	14		O			⊕		⊕	⊕	⊕	+		O						⊕	⊕	+ ^A^	⊕	⊕	
	15					O		+	⊕	⊕	+	⊕		⊕					⊕	+ ^B^	+ ^A^	⊕	O	O ^C^ pharyngeal tonsils, + ^A^ pleura
	16 ^D^								+	⊕	+													⊕ inj. site trachea
1 left mandibular LN							6 left medial retroph. LN							11 liver				16 bone marrow				21 mesenteric LNs		
2 right mandibular LN							7 right medial retroph. LN							12 hepatic LNs				17 heart				22 ileocolic LNs		
3 left parotid LN							8 left tracheobronchial LN							13 spleen				18 JPP				23 other sites		
4 right parotid LN							9 mediastinal LNs							14 kidney				19 IPP						
5 palatine tonsil							10 lung							15 renal LNs				20 other GALT						

O isolation, no lesion, + lesion, no isolation, ⊕ lesion and isolation, ^A^ only morphological examination, isolation not done, ^B^ culture not assessable because of contamination, ^C^ only isolation, morphological examination not performed, ^D^ intratracheal inoculation.

## Data Availability

The data presented in this study are available on request from the corresponding author.

## Supplementary Materials

The following supporting information can be downloaded at: https://www.mdpi.com/article/10.3390/ijms25189799/s1 and at: https://doi.org/10.5281/zenodo.13123137.
