# Supplementary material for: Challenge Dose Titration in a Mycobacterium bovis Infection Model in Goats
Source: Int J Mol Sci. 2024 Sep 10;25(18):9799. doi: 10.3390/ijms25189799 (PMC11431947; doi:10.3390/ijms25189799)
Supplement: Supplementary file 1 [file ijms-25-09799-s001.zip › ijms-3150376-supplementary.pdf]

International Journal of *Molecular Sciences*

MDPI

Supplementary Materials: The following supporting information can be downloaded at: www.mdpi.com/xxx/s1,

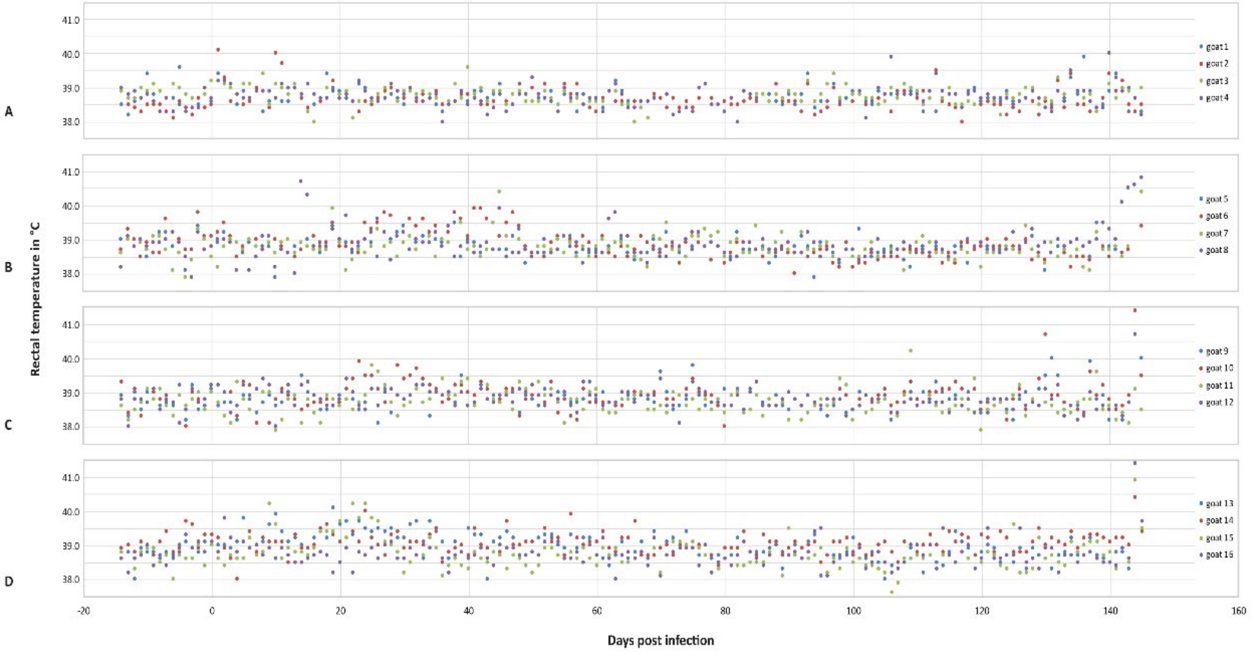

Figure S1 A, B, C, D. Daily body temperature before and after inoculation (0 dpi) in mock-inoculated controls (A) and goats inoculated with the LD (B), MD (C) and HD (D) of *M. bovis*. Individual animals of each group are color coded. Increased fluctuation and mild fever are seen in all goats inoculated with *M. bovis* between 20 dpi and 50 dpi and in the HD-group until 140 dpi. Fever peaks occur in all goats inoculated with *M. bovis* after the application of SICCT at 143 dpi.

Table S1. Number and volume of pulmonary lesions detected by CT-Scans as hyperdense lesions or caverns

Group	Goat	Number of lesions					Volume of lung in cm^3	Volume of lung lesions	
		Micro-nodules < 5 mm	Consolidations > 5 mm: unicentric mineralization	Consolidations > 5 mm: multicentric mineralization	Caverns	Sum		in cm^3	in %
mock	1	0	0	0	0		2495.3	0	0
mock	2	0	0	0	0	0	1646.2	0	0
mock	3	0	0	0	0		2694.5	0	0
mock	4	0	0	0	0		2469.3	0	0
mean mock-inoculated		0	0	0	0		2326.4	0	0
low dose	5	34	7	3	0		2415.6	9.8	0.41
low dose	6	34	1	2	0	120	2499.8	33.7	1.35
low dose	7	10	0	0	0		2695.3	7.6	0.28
low dose	8	21	5	3	2		3255.1	1117.0	34.31
mean low dose		24.7	3.2	2	0.2		2716.5	292.0	9.09
medium dose	9	56	6	5	0		3105.2	45.1	1.45
medium dose	10	11	15	3	0	226	2943.4	66.1	2.24
medium dose	11	47	1	5	0		2423.2	45.2	1.86
medium dose	12	76	0	1	0		2297.5	12.8	0.56
mean medium dose		47.7	5.5	3.5	0		2692.3	42.3	1.53
high dose	13	176	5	8	0		2189.9	39.7	1.81
high dose	14	42	3	3	2	266	2984.3	298.3	9.99
high dose	15	15	6	8	1		3122.3	463.1	14.83
high dose it[a]	16	0	0	0	0		2639.1	0	0
mean high dose		77.7	4.7	6.3	1		2725.6	200.3	6.66

[a]intratracheal application of inoculum

Table S2. Scoring of clinical signs

**Parameters**	**Features**	**Score**
general condition (posture/behavior)	lively and alert	0
	lies a lot, somewhat sluggish	1
	does not stand for more than 1-2 minutes	2
	apathetic, unable to stand up	3
skin and hair	normal	0
	focal/multifocal alopecia	1
	decubitus	2
nasal and occular secretion	no	0
	little, watery	1
	little, slimy/flaky	2
	much, purulent	3
conjunctiva/oral mucosa	normal	0
	anemic	1
	cyanotic	2
	slightly reddened	1
	red	2
	icteric	1
	surface alterations	1
	dry	1
breathing pattern	normal	0
	mild dyspnoe	1
	moderate dyspnoe	2
	severe dyspnoe	3
spontaneous coughing	no	0
	sporadic	1
	occasionally	2
	frequently	3
inducible coughing	no	0
	sporadic	1
	paroxysmal	2
quality of cough	dry	1
	moist	2
	productive (sputum)	3
appetite	normal	0
	drinks/eats listlessly and less than normal	1
	no food intake	2
rumination	yes	0
	no	1
fecal consistency	normal	0
	soft	1
	pasty	2
	diarrhoe	3
	frothy diarrhea	4
legs	normal	0
	mono- or polyarthritis of 1-3 joints	1
	polyarthritis of > 3 joints	2
size of peripheral lymph nodes	normal	0
	slightly enlarged	1
	greatly enlarged	2
painfulness of peripheral lymph nodes	no	0
	yes	1
